# Label-free SERS study of galvanic replacement reaction on silver nanorod surface and its application to detect trace mercury ion

**DOI:** 10.1038/srep19650

**Published:** 2016-01-21

**Authors:** Yaohui Wang, Guiqing Wen, Lingling Ye, Aihui Liang, Zhiliang Jiang

**Affiliations:** 1 Key Laboratory of Ecology of Rare and Endangered Species and Environmental Protection of Ministry Education, Guangxi Key Laboratory of Environmental Pollution Control Theory and Technology, Guangxi Normal University, Guilin 541004, China

## Abstract

It is significant to explore a rapid and highly sensitive galvanic replacement reaction (GRR) surface enhanced Raman scattering (SERS) method for detection of trace mercury ions. This article was reported a new GRR SERS analytical platform for detecting Hg(II) with label-free molecular probe Victoria blue B (VBB). In HAc-NaCl-silver nanorod (AgNR) substrate, the molecular probe VBB exhibited a strong SERS peak at 1609 cm^−1^. Upon addition of Hg(II), the GRR occurred between the AgNR and Hg(II), and formed a weak SERS activity of Hg_2_Cl_2_ that deposited on the AgNR surfaces to decrease the SERS intensity at 1609 cm^−1^. The decreased SERS intensity 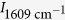 was linear to Hg(II) concentration in the range of 1.25–125 nmol/L, with a detection limit of 0.2 nmol/L. The GRR was studied by SERS, transmission electron microscopy and other techniques, and the GRR mechanism was discussed.

The toxicity of mercury is in the first place among the toxic heavy metals, and it is easy to accumulate in human body and animals even very low concentration through the food chain^1^,^2^. Inorganic mercury can bind with protein, and inhibit the enzyme’s activity to retard cell metabolism^3^. At present, the methods of detecting mercury ions include colorimetry^4^,^5^,^6^, atomic fluorescence spectrometry (AFS)^7^,^8^,^9^, gas liquid chromatography-mass spectrometry (GC-MS)^10^,^11^,^12^, atomic absorption spectrometry (AAS)^13^,^14^,^15^, resonance Rayleigh scattering (RRS)^16^,^17^, SERS^18^,^19^. The AAS, AFS and GC-MS methods were general and sensitive, but their process was too complex and high cost. Colorimetric method was simple and economic, but it was not sensitive. RRS method is simple, rapid and sensitive, but the selectivity remains to be improved. Therefore, it is significance to establish a highly sensitive and selective analytical method to detect mercury ions.

Silver nanoparticles possess advantages of low-cost, high molar extinction coefficient and its aggregates are of low molar extinction coefficient and strong SERS effect. It provides the foundation for their applications^20^,^21^,^22^. SERS is not only detected solely trace analyte which adsorbed on the nanosurface especially nanosilver, but also present rich information about molecular structure^23^. It is a powerful spectral technique with non-destructive and ultra-sensitive characterization, even detecting single molecular^24^. Nowadays, looking for the stable and highly sensitive nanosol SERS substrate, such as nanogold and nanosilver and its application have attracted much attention in chemistry, environment and clinical medicine fields. Liu *et al.*^25^ reported that Hg^2+^ can combine with C=O and −NH_2_ molecule probe and lead to SERS signals quenched on the Au/Ag alloy surface. Cui *et al.*^26^ developed a SERS-active platform by employing the oligonucleotide-functionalized magnetic silica sphere (MSS)@Au nanoparticles (NPs) and its aptamer reaction. Ray *et al.*^27^ utilized popcorn shaped gold nanomaterials that was protected by tryptophan as SERS substrate for rapid, easy and highly selective recognition of Hg(II) ions at 5 ppb level in aqueous solution. According to whether or not use molecular probe as label, SERS is divided into labeled SERS and label-free SERS techniques. Labeled SERS technique has advantages of rapidity, sensitivity and specificity^28^. Although this SERS-based detection technique provides a sensitive method for immobilized immunocomplexes, it has several drawbacks^29^. First, an extended incubation time was required on molecular diffusion near the surface. Second, all of the immunoreagent components should be immobilized on the surface of a solid substrate in air. Third, the exposure of components to air seriously reduced their activity. The last, it is easy occurring nonspecific adsorption that lead to false positive and background signal rising^30^. Label-free SERS technique can detect directly the Raman signal of analyte and detect indirectly analyte by the Raman signal of dye molecular probe. As another application of SERS, label-free SERS techniques have begun to emerge as potential methods using Raman optical fingerprint. It has advantages of simplicity, rapidity, directly using the characteristics of sample’s Raman signal without extra label sample processing. And best of all, it can avoid the damage to the samples. Hoan *et al.*^31^ have demonstrated a novel label-free DNA biosensor based on molecular sentinel immobilized on nanowave/metal film over nanosphere substrate. This DNA biosensor is relatively easy to fabricate at low-cost and can specifically detect a complementary target DNA. Li *et al.*^32^ proposed an iodide-modified Ag nanoparticles method for label-free detection of proteins. Aoune *et al.*^33^ reported a simple, label-free detection scheme for DNA hybridization based on surface-enhanced Raman spectroscopy. Yang *et al.*^34^ had successfully applied Ag nanoparticles for label-free detection of thiram on apple peels, and demonstrated their potential for use as a SERS-based on-site detection method for various pesticides. However, label-free SERS techniques used in detection of heavy metal ions were rarely.

Galvanic replacement reaction (GRR), which a more reactive metal is etched by a less reactive one, happened usually in several minutes^35^. It is particularly interesting due to its high tunability and the possibility to study the intricacies of alloying and dealloying in metallic nanostructures^36^. In addition, GRR provides a remarkably simple and versatile route to metal nanostructures with controllable hollow interiors and porous walls^37^. Xia *et al.*^38^ used Ag as a template coupling with Au^3+^ redox reaction to prepare Au hollow structure, and the reaction mechanisms were also explained. Li *et al.*^39^ reported a strategy that anti-GRR occurred between ultra-small ssDNA-templated silver nanoclusters and Cu(II) ions to synthetize Ag/Cu alloy that was monitored by light scattering technique. Ye *et al.*^40^ have obtained hetero-structured nanotubes which have high electrocatalytic activities by GRR of between Ag/AgCl core–shell nanowires and H_2_PtCl_6_. Jiang *et al.*^41^ reported a highly sensitive and selective SERS substrate by GRR. Li *et al.*^42^ presented a facile and general GRR route to produce silver and gold dendrites as well as other metal hierarchical micro/nanostructures (Cu, Pt, Pd, Ni and Co) on commercial aluminum foil in the presence of NaF or NH_4_F. The obtained silver and gold dendrites showed significantly SERS signals of a self-assembled monolayer of 2-naphthalenethiol and 4-mercaptobenzoic acid in aqueous solution. There are rare reports about GRR used in SERS quantitative analysis. Qin *et al.*^43^ reported a strategy to complement the GRR between Ag nanocube and HAuCl_4_ with co reduction by ascorbic acid (AA) for the formation of Ag-Au hollow nanostructures to detect 1,4-benzenedithiol SERS signal. The GRR between As nanoparticles and Au(III) ions has been reported for the first time by Pal *et al.*^44^. The potential of such an assembly was further exploited for SERS detection of Rhodamine 6 G, 4-mercaptopyridine and 4-aminothiophenol. An effective SERS substrate was fabricated by Fu *et al.*^45^ via Ag dendrites on Al foil by GRR with [Ag(NH_3_)_2_]Cl for detecting biomolecules like folic acid, DNA and RNA which used as molecular probe directly in aqueous solution. The detection concentration for the three biomolecules have reached the level of 1 pmol/L, the symmetric silver dendrites can potentially be employed as effective SERS sensors for label-free and ultrasensitive biomolecule detection. Yi *et al.*^46^ synthesized dendritic Ag-Pd bimetallic nanostructures on the surface of Cu foil via multistage GRR of Ag dendrites in a Na_2_PdCl_4_ solution to detect fluorescent rhodamine 6G (Rh6G) molecules at a concentration of 10^−6^  mol/L. A transparent Ag thin film composed of vertically aligned and single crystalline silver nanopetals with uniform distribution were fabricated by GRR^47^, the Raman intensity showed concentration-dependent behavior following the Freundlich equation, with a detection limit of 500 pmol/L crystal violet. As far as we know, there are no reports about the label-free SERS quantitative analysis of Hg(II) via the nanosurface GRR. In this article, the AgNR exhibited strong SERS activity, but the Ag/Hg_2_Cl_2_ nanoparticles from the GRR of Hg(II)-AgNR had very low SERS activity that caused SERS quenching. Thus, a simple, rapid, sensitive and selective SERS quantitative method was established for detection of Hg(II) in aqueous solution.

## Results

### SERS spectra in nanosilver sol substrate

**Figure 1:**
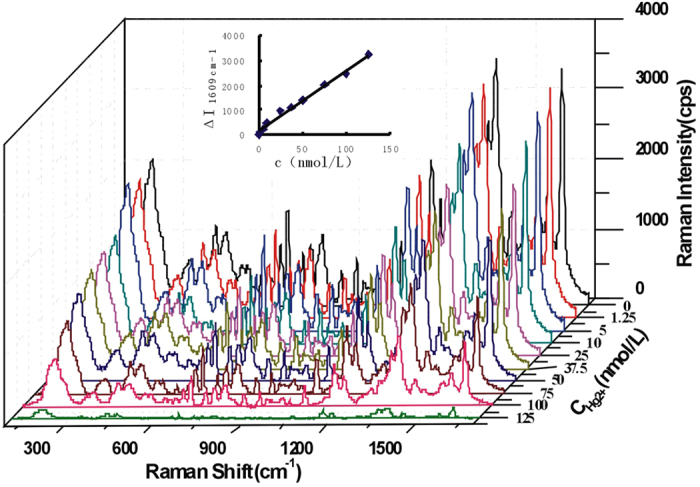
SERS spectra of Hg(II)-AgNR-VBB system. 1 × 10^−4^ mol/L AgNR-75 mmol/L HAc-20 mmol/LNaCl-1.5 × 10^−7^ mol/L VBB-Hg(II).

In 75 mmol/L HAc solution containing high concentrations of NaCl, VBB was adsorbed on the surface of nanosilver such as AgNR, AgNC, AgNT and AgNP aggregates with strong SERS activity and exhibited 12 SERS peaks (Table 1)^48^. Upon addition of trace Hg(II), the GRR took place between silver atoms and Hg(II) to form a larger size core-shell composite nanoparticles Ag_core_/Hg_2_Cl_2shell_ with low SERS activity that lead to SERS signals quenching. Therefore, with the increase of the Hg(II) concentration, the SERS intensity decreased linearly at 1609 cm^−1^ (Fig. 1, Fig. S1, S2, S3).

### SERS spectra of different molecular probe

In 75 mmol/L HAc solution, the diphenyl methane dye of VBB, triphenylmethane dye such as Rh6G, RhS and RhB, safranine dye of ST, acridine dye of AR, PTD and TPPS as molecular probes were examined that could be adsorbed on the surface of AgNR aggregates with strong SERS peaks (Table S1). Upon addition of trace Hg(II), the GRR took place and their SERS intensity decreased linearly (Fig. 1, S4–S10). The SERS spectra of 8 molecular probes were compared when AgNR sol was used as SERS active substrate, and the assignment of SERS peaks were analyzed in details (Table S1).

### Transmission electron microscopy (TEM)

**Figure 2:**
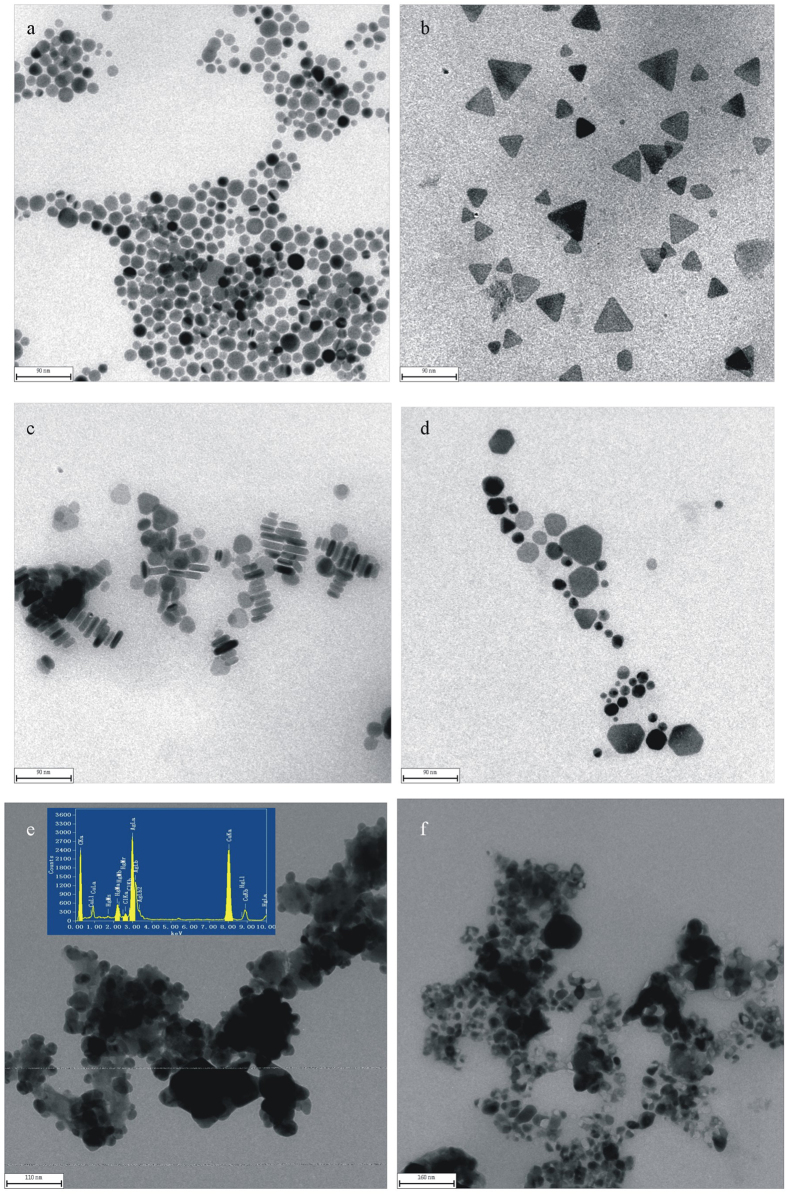
TEMs of the silver nanoparticles (**a**) AgNP; (**b**) AgNT; (**c**) AgNR; (**d**) AgNC; (**e**) 1 × 10^−4^ mol/L AgNR-75 mmol/L HAc-0.25 μmol/L Hg(II); (**f**) 1 × 10^−4^ mol/L AgNR-75 mmol/L HAc-2.5 μmol/L Hg(II).

The TEMs of AgNP, AgNT, AgNR and AgNC showed in Fig. 2a–d. In Fig. 2b, silver nanoparticles are most triangle with the side length between 18–72 nm. Compared with AgNT, the size of AgNR was smaller with a diameter of 9 nm and a length of 18–45 nm, in addition there are spherical nanosilvers (Fig. 2c). The TEM of AgNR-Hg(II) system show that GRR would take place when trace Hg(II) was added. The reaction ended until the Ag template completely dissolved and obtained nanomercury with hollow structure (Fig. 2f). The energy spectra of AgNR-HgCl_2_ system were recorded (Fig. 2e), in which Ag element exhibited three peaks at 2.984, 3.2 and 3.8 keV, Hg exhibited three peaks at 1.7, 2.159 and 10 keV, and Cu was ascribed to the copper network that was used for the loading sample. The results indicated that the bimetallic nanostructures were made up of Ag atoms and Hg atoms.

### Influence of the reaction medium

**Figure 3:**
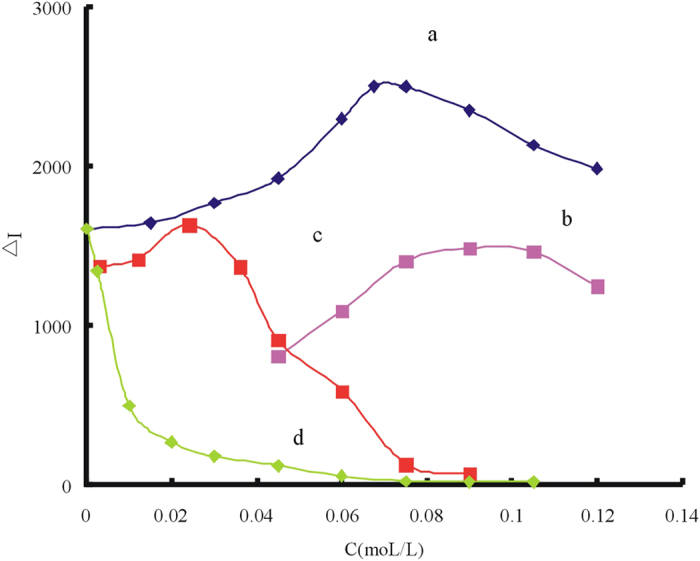
Influence of the reaction medium (**a**) HAc -1 × 10^−4^ mol/L AgNR -75 nmol/L Hg(II)-20 mmol/L NaCl-1.5 × 10^−7^mol/L VBB; (**b**) HCl -1 × 10^−4^mol/L AgNR -75 nmol/L Hg(II)-20 mmol/L NaCl-1.5 × 10^−7^mol/L VBB; (**c**) H_2_SO_4_ -1 × 10^−4^mol/L AgNR -75 nmol/L Hg(II)-20 mmol/L NaCl-1.5 × 10^−7^mol/L VBB; (**d**) NaOH -1 × 10^−4^mol/L AgNR -75 nmol/L Hg(II)-20 mmol/L NaCl-1.5 × 10^−7^mol/L VBB.

The effect of reaction medium on the GRR was examined (Fig. 3). When HCl was used as medium, the GRR would take place and the nanosurface Ag atom would lose electrons to form Ag^+^ and dissolved into the solution. The Ag^+^ reacted with Cl^−^ to from white precipitation AgCl. With the increase of concentration of Hg(II), the solution gradually become turbid. The AgCl precipitation affected the stability and the sensitivity reduced compared with water medium, so HCl was not chosen as medium. When H_2_SO_4_ was used as medium, nanosilver gathered because the acid was too strong, it was not beneficial to react fully between Hg(II) and Ag, so H_2_SO_4_ was not chosen as medium too. When NaOH was used as medium, in the same way, Ag^+^ would react with OH^−^ to generate precipitation AgOH which is very unstable and easily broken down into Ag_2_O black precipitation. With the increase of concentration of Hg(II), it can be observed trace black precipitate at the bottom and the sensitivity reduced compared with water medium too, so NaOH was not chosen as medium. When HAc was used as medium, the effect of HAc concentration was examined and the VBB SERS intensity was improved greatly on the surface of the silver because the active sites can be improved in the weak acid environment^49^. The results showed that the Δ*I* value reached its maximum when the concentration was 75 mmol/L. In conclusion, a 75 mmol/L of HAc solution was chosen as medium.

### Effect of the reducing agent

**Figure 4:**
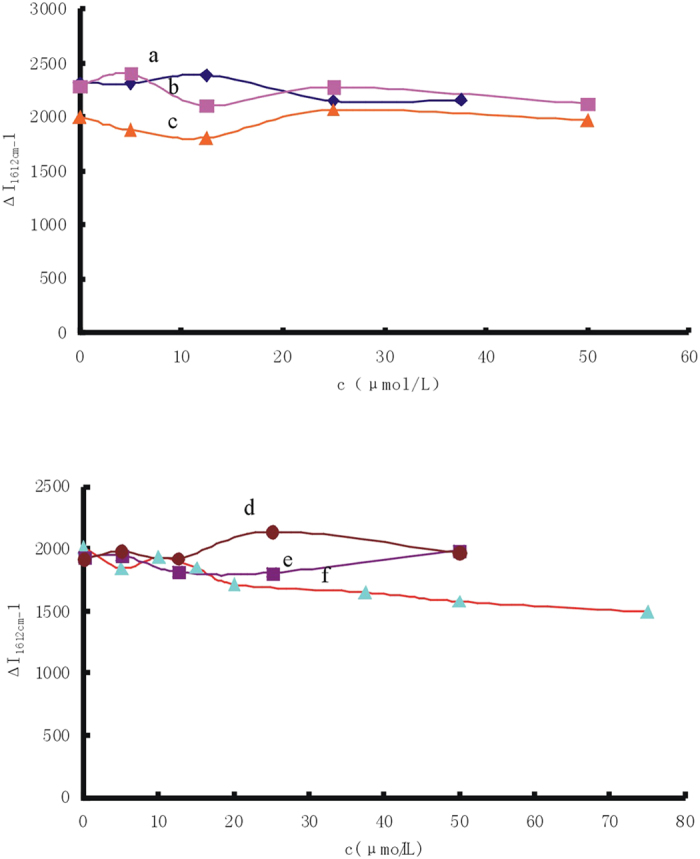
Influence of reducing agent concentration (**a**) 1 × 10^−4^mol/L AgNR-75 mmol/L HAc-75 nmol/L Hg(II) -20 mmol/LNaCl -1.5 × 10^−7^mol/L VBB-NaBH_4_; (**b**) 1 × 10^−4^mol/L AgNR -75 mmol/L HAc-75 nmol/L Hg(II) -20 mmol/LNaCl -1.5 × 10^−7^mol/L VBB-Vc; (**c**) 1 × 10^−4^mol/L AgNR -75 mmol/L HAc-75 nmol/L Hg(II) -20 mmol/LNaCl -1.5 × 10^−7^mol/L VBB- hydrazine hydrate; (**d**) 1 × 10^−4^mol/L AgNR -75 mmol/L HAc- 75 nmol/L Hg(II) -20 mmol/LNaCl -1.5 × 10^−7^mol/L VBB - hydroxylamine hydrochloride; (**e**) 1 × 10^−4^mol/L AgNR -75 mmol/L HAc-75 nmol/L Hg(II) -20 mmol/LNaCl -1.5 × 10^−7^mol/L VBB-H_2_O_2_; (**f**) 1 × 10^−4^mol/L AgNR -75 mmol/L HAc-75 nmol/L Hg(II) -20 mmol/LNaCl -1.5 × 10^−7^mol/L VBB-Na_2_SO_3_.

In the process of preparation of nanosilver, the effect of reductants such as NaBH_4_, H_2_O_2_ and hydrazine hydrate was studied. Although the reductants will break down after a certain period of time, in order to rule out the possibility of Hg(II) reduced by the residual reductant, different reducing agents such as NaBH_4_,Vc, hydrazine hydrate, hydroxylamine hydrochloride, H_2_O_2_ and Na_2_SO_3_ were examined respectively. Results showed that only Na_2_SO_3_ inhibited the GRR reaction when the concentration was greater than 2 × 10^−5^ mol/L because a strong reducing agent competed with the galvanic replacement between Ag and Hg(II)^50^. The SERS signals were not changed with the increase of concentration of the other reducing agents (Fig. 4). Thus the formation of Ag_core_/Hg_2_Cl_2shell_ was not reduced by this reductant.

### Screen of nanosilver SERS substrates

**Figure 5:**
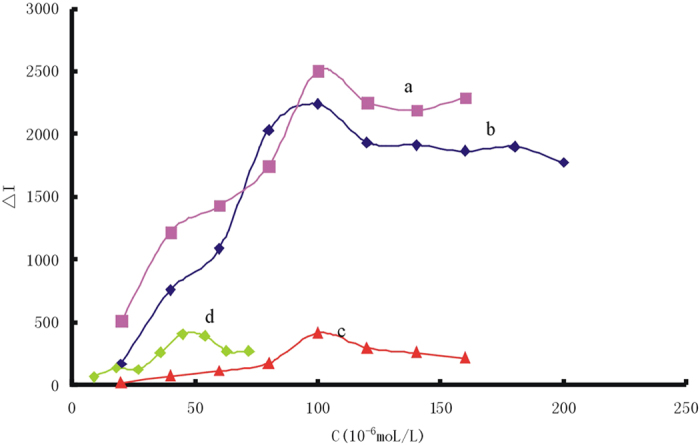
Effect of nanosilver concentration (**a**) AgNR-75 mmol/L HAc-75 nmol/L Hg(II) -20 mmol/LNaCl-1.5 × 10^−7^mol/L VBB; (**b**) AgNC-75 mmol/L HAc-75 nmol/L Hg(II) −20 mmol/LNaCl -1.5 × 10^−7^mol/L VBB; (**c**) AgNT-75 mmol/L HAc -75 nmol/L Hg(II) -20 mmol/L NaCl-1.5 × 10^−7^mol/L VBB; (**d**) AgNP-75mmol/L HAc-75 nmol/L Hg(II)-20 mmol/L NaCl-1.5 × 10^−7^mol/L VBB.

The effect of four kinds nanosilver sol substrate was examined when VBB was used as SERS molecular probe (Fig. 5). The results showed that the Δ*I* value reached maximum when the concentration of AgNP was 4.5 × 10^−5^mol/L and the concentration of AgNT, AgNR and AgNC was 1.0 × 10^−4^ mol/L. Thus, a 4.5 × 10^−5^mol/L AgNP, and 1.0 × 10^−4^ mol/L AgNT, AgNR and AgNC were chosen. The AgNR sol substrate was most sensitive to detect Hg(II).

### Screen of SERS molecular probes

**Figure 6:**
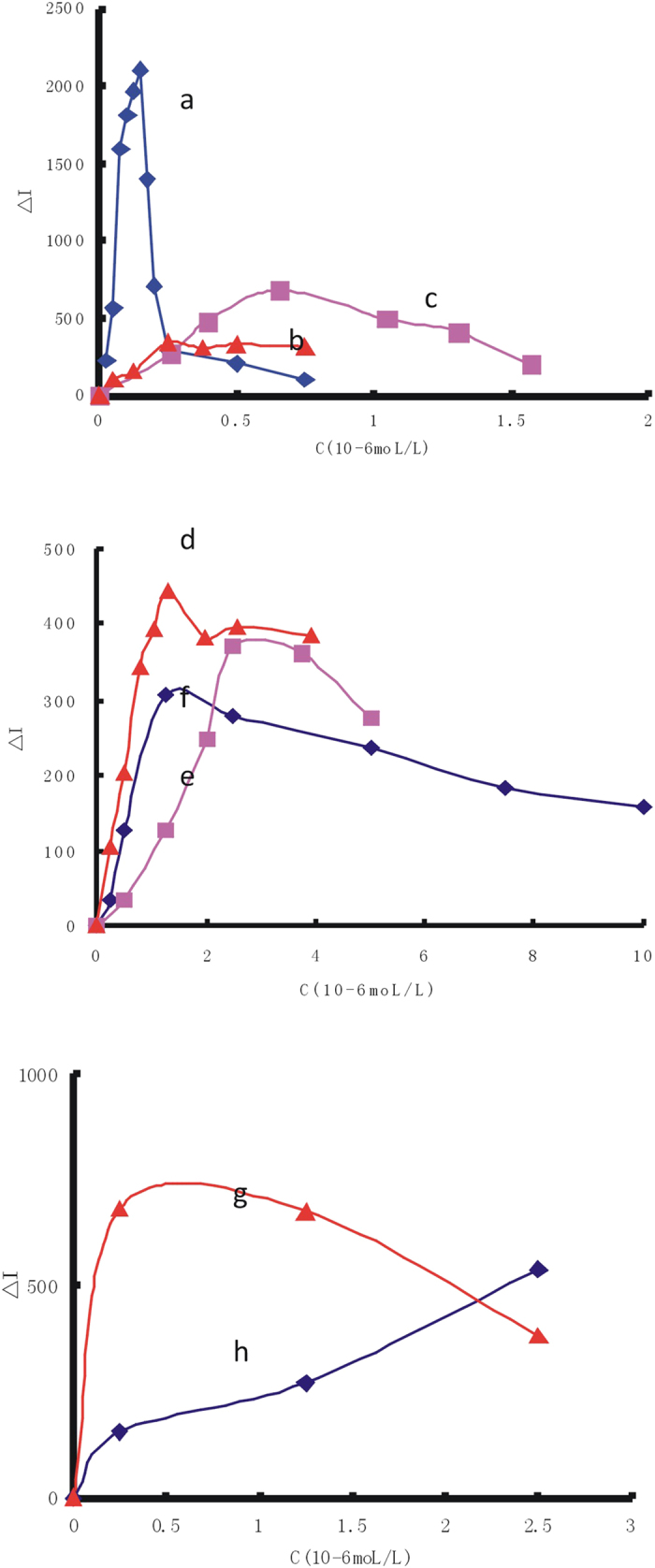
Effect of molecular probe concentration (**a**) 1 × 10^−4^mol/L AgNR-75 mmol/L HAc-75 nmol/L Hg(II) -20 mmol/LNaCl-VBB; (**b**) 1 × 10^−4^mol/L AgNR-75 mmol/L HAc-75 nmol/L Hg(II)-Rh6G; (**c**) 1 × 10^−4^mol/L AgNR-75 mmol/L HAc-75 nmol/L Hg(II)-20 mmol/LNaCl-ST; (**d**) 1 × 10^−4^mol/L AgNR -75 mmol/L HAc-75 nmol/L Hg(II)- RhS; (**e**) 1 × 10^−4^mol/L AgNR -75 mmol/L HAc- 75 nmol/L Hg(II)-AR; (**f**) 1 × 10^−4^mol/L AgNR-75 mmol/L HAc- 75 nmol/L Hg(II)-RhB; (**g**) 1 × 10^−4^mol/L AgNR-75 mmol/L HAc-75 nmol/L Hg(II)-PTD; (**h**) 1 × 10^−4^mol/L AgNR-75 mmol/L HAc-75 nmol/L Hg(II)-TPPS.

The effect of molecular probes was examined when AgNR was used as SERS substrates (Fig. 6). The results showed that the Δ*I* value reached maximum when 1.5 × 10^−7^ mol/L VBB, 2.5 × 10^−7^ mol/L ST, 6.5 × 10^−7^mol/L Rh6G, 1.3 × 10^−6^ mol/L RhS, 1.25 × 10^−6^mol/L RhB, 2.5 × 10^−6^mol/L AR and 2.5 × 10^−6^ mol/L PTD were used respectively. The SERS intensity of TPPS would be interfered by its fluorescence. So 1.25 × 10^−6^ mol/L TPPS was chosen. Summary above, VBB was chosen to detect Hg(II) because it had the highest sensitivity among the molecular probes.

### Effect of foreign substances

According to the procedure, the effect of foreign substances on the determination of 100 nmol/L Hg(II) was tested, with a relative error within ±10%. Results showed that 10 μmol/L Zn^2+^, Mg^2+^, Mn^2+^, Pb^2+^, Na^+^, K^+^, Ca^2+^, Al^3+^, Fe^3+^, Ag^+^, Cu^2+^, Br^−^ and I^−^, 8 μmol/L SeO_3_^2−^, 5 μmol/L Bi^3+^, 4 μmol/L TeO_4_^−^, BSA and L-cystine, 1 μmol/L HSA and L-lysine did not interfere with the determination, which indicated that this GRR SERS method had good selectivity (Table S2).

### Analytical feature and application

Under the optimal conditions, for the Hg(II)-nanosilver-VBB system, four kinds of nanosilver sols were chosen as substrates respectively, the SERS intensity for different Hg(II) concentrations(C) were recorded and the working curves were drawn according to the relationship between C and their corresponding Δ*I* values. The results showed that AgNR had the highest sensitivity among the four kinds of nanosilver (Table 2). From the comparison of reported assays for SERS detection of Hg(II)^51^,^52^,^53^ (Table S3), this GRR method has the advantages of simplicity, rapidity, high sensitivity and specificity. Thus, it can be used as a new method for rapid detection of Hg(II). For the Hg(II)-AgNR-molecular probe system, 8 kinds of molecular probes were considered for detection of Hg(II), the SERS intensity for different Hg(II) concentrations(C) were recorded and their working curves were drawn according the relationship between C and their corresponding Δ*I* values. The Fig. 6 showed that the VBB had the highest sensitivity among the 8 molecular probes (Table 3). In the sample solution, the coexistence of metal ions are mainly sodium, potassium, calcium, magnesium, zinc, aluminum, titanium, silicon and iron which are not interfere with the determination of Hg(II). There are no gold, palladium and platinum ions in the cosmetics samples, although they interfered with the determination and had the relative errors of 18%, 20% and 15%, respectively, when they were in the same concentration level as mercury ions. So the determination results are credible. The Hg contents in six kinds of cosmetic sample have been detected by the SERS method. Then, a known amount of Hg(II) was added into the sample to obtain the recovery. The results (Table S4) showed that the relative standard deviation was in the range of 0.58–3.72%, and the recovery was in the range of 95.40–107.56% that indicated this method was accuracy. The US Food and Drug Administration (FDA) limits the amount of mercury in cosmetic products to a trace amount unavoidable under good manufacturing practice, 1 ppm (1*μ*g/g), except in those products intended for use around the eye, which it limits to 65 ppm^54^,^55^,^56^. According to the standards, the Hg content in the detected cosmetic products up to grade.

## Discussion

### Analytical principle

As we know, GRR takes place between a suspension of nanoscale metal templates and a salt precursor containing a relatively less active metal. The driving force of the GRR is the electrical potential difference between two metals. Generally, a GRR can be split into two half reactions, the oxidation/dissolution of a metal at the anode, and the reduction/deposition of the ions and a second metal at the cathode. It is critical that the electrochemical potential of the metal ions is higher than that of the solid metal to occur this reaction^57^. Since the standard reduction potential (SRP) of Hg^2+^ /Hg^0^ (0.851 V *vs* Standard Hydrogen Electrode, SHE) and Hg^2+^ /Hg_2_^2+^ (0.920 V *vs* SHE) are higher than that of Ag^+^ /Ag (0.7996 V *vs* SHE), the surface of Ag nanostructures suspended in the solution can be oxidized by Hg(II), in which produce Ag^+^, Hg_2_^2+^ and Hg^0^ according to reaction (1) and (2)^58^, but the SRP of Ag^+^ /Ag (0.7996 V *vs* SHE) is higher than that of Hg_2_^2+^ /Hg^0^ (0.7973 V *vs* SHE) that makes Ag^+^ oxidizing Hg^0^ to Hg_2_^2+^ according to reaction (3). In conclusion, the final products were Ag^+^ and Hg_2_^2+^. The relevant equations between Hg and Ag are shown below,


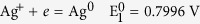



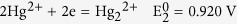



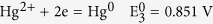



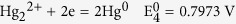



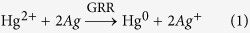



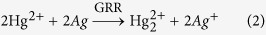



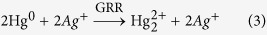


**Figure 7:**
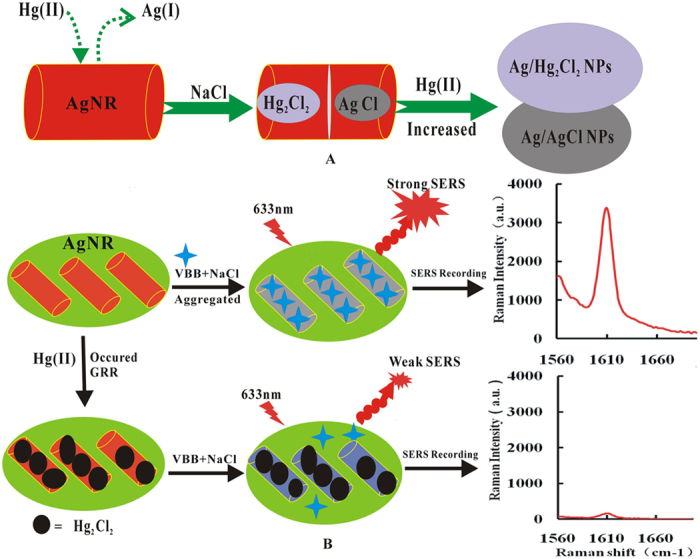
Schematic illustration of the Hg(II)-AgNR GRR monitored by SERS technique.

In 75 mmol/L HAc solution containing high concentration of NaCl, the SERS molecular probe was adsorbed on the surface of AgNR aggregates sol with strong SERS signal. Upon addition of trace Hg(II), it would be adsorbed on the surface of AgNR. Ag atom would lose electrons to form Ag^+^ and the Hg(II) got electrons to form Hg_2_Cl_2_ on the outer layer of AgNR because of the GRR of Ag atoms and Hg(II) (Fig. 7A), meanwhile the hydrophobic Hg_2_Cl_2_ and AgCl molecules formed on the surfaces. With the reaction going on, Ag atom continues losing electrons to form Ag^+^ that dissolved into the solution and formed the larger Ag pores. The SRP of Ag^+^ /Ag was higher than that of Hg_2_^2+^/Hg^0^, and this made Ag^+^ oxidize Hg^0^ to Hg_2_^2+^, and the Hg(II) form Hg_2_Cl_2_ continuous precipitation. The final products were larger size core-shell composite nanoparticles with the silver nanoparticles as core and Hg_2_Cl_2_ as shell (Ag_core_-Hg_2_Cl_2shell_). The reaction ended until the Ag template completely dissolved and obtained hollow structure Ag_core_/Hg_2_Cl_2shell_ (Fig. 7A). The AgNR was one of the strongest SERS substrates but the nanomercury was weaker than AgNR. Therefore, with the increased concentration of Hg(II), the amount of core-shell composite nanoparticles Ag_core_-Hg_2_Cl_2shell_ increased and lead to the SERS intensity decrease (Fig. 7B). Base on this ground, a simple, rapid, sensitive and selective GRR label-free SERS method was established to detect trace Hg(II) in aqueous solution.

### Indentifying GRR product of Hg(II)

**Figure 8:**
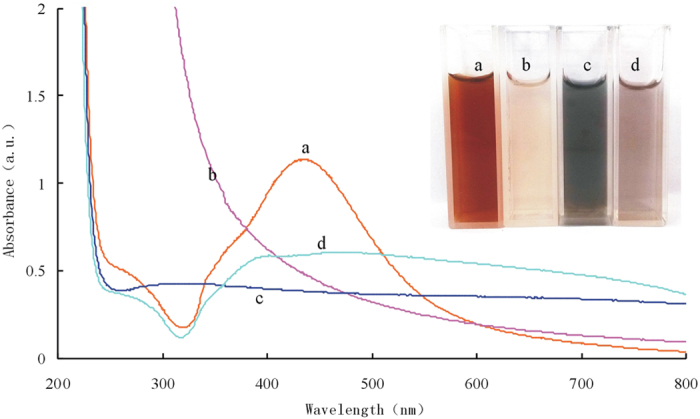
UV spectra of the AgNR-Hg(II) system (**a**) 4 × 10^−4^mol/L AgNR -0.3 mol/L HAc; (**b**) 4 × 10^−4^mol/L AgNR -0.3 mol/L HAc -0.1 mmol/L Hg(II); (**c**) 4 × 10^−4^mol/L AgNR -0.3 mol/L HAc -0.1 μmol/L Hg(II)-0.24 mol/L NH_3_.H_2_O; (**d**) 4 × 10^−4^mol/L AgNR -0.3 mol/L HAc-0.24 mol/L NH_3_.H_2_O.

In order to identifie directly the final product is Hg^0^ or Hg_2_^2+^ with the naked eye, the concentration of reactant are increased multiply. The AgNR exhibited two SPR absorption peaks at 312 nm and 430 nm (Fig. 8a). It would take place GRR with Hg(II) to exhibit turbid white solution (Fig. 8b). But it was not observed black elemental mercury precipitation in solution. So this phenomenon showed clearly that there were no elemental mercury in the reaction products and Hg^0^ has been oxidized by Ag^+^ because the SRP of Ag^+^/Ag (0.7996 V *vs* SHE) is higher than that of Hg_2_^2+^/Hg^0^ (0.7973 V *vs* SHE). The white turbid solution was the mixture of Ag_core_/(Hg_2_Cl_2_)_shell_ and a part of Hg_2_Cl_2_ in solution. The UV absorption decreased since the Hg(II) oxidized the AgNR to colorless Ag^+^, the SPR absorption peak of AgNR disappearing intimated that AgNR has been oxidized by Hg(II). In order to verify the final product of GRR reaction is Hg_2_^2+^, a 0.24 mol/L NH_3_.H_2_O was added into the solution. Figure 8b obtained a black solution due to the reaction between ammonia and Hg_2_Cl_2_ generate white precipitate Hg(NH_2_)Cl and black precipitate mercury. The Fig. 8c solution without SPR peak showed that there was no AgNR in the solution. Therefore it can be ruled out the possibility of the black solution being the excess AgNR aggregates, but the reaction of ammonia and Hg_2_Cl_2_ generated black precipitate mercury. In conclusion, the final products were Ag^+^ and Hg_2_^2+^.

## Methods

### Apparatus and reagents

A model of DXR smart Raman spectrometer (Thermo Company, USA) was used, with a laser wavelength of 633 nm, power of 2.5 mW, average number of scanning of 2, collect exposure time of 7.5 s, exposure time of 1.0 s, sample exposures of 2. A model of H-800 transmission electron microscopy (Hitachi LTD., Japan) with point spacing of 0.45 nm, lattice resolution of 0.204 nm, accelerating voltage of 200KV and tilt angle of ±25^o^, and a model of TU-1901 double-beam UV-Vis spectrophotometer (Beijing Purkinje General Instrument Co. Ltd., China) were used.

A 500 μL 0.3 mol/L HAc, 500 μL AgNR or AgNC, AgNT and AgNP solution, a certain amount of HgCl_2_ solution were added into a 5 mL marked-test tube respectively and mixed well. Different molecular probe such as VBB, ST, Rh6G, RhS, RhB, AR, MH, FL, PTD and TPPS was added in the mixture respectively, and diluted to 2 mL. If VBB, VB4R, ST were used as SERS molecular probe, a 20 mmol/L NaCl was added. The mixture was transferred to a 1 cm quartz cell, the SERS intensity and the corresponding blank value *I*_*0*_without HgCl_2_ were recorded. The value of Δ*I* = *I*_*0*_ –*I* was obtained.

### Reagents

Hg^2+^ standard solution was prepared as follows, a 0.2715 g HgCl_2_ was dissolved in water, diluted to 100 mL with water to obtain 10 mmol/L Hg^2+^ stock solution. A 0.3 mol/L HAc solution, 10 μmol/L Victoria blue (VBB), 0.1 mmol/L rhodamine B (RhB), 52.3 μmol/L rhodamine S (RhS), 52.3 μmol/L rhodamine 6 G (Rh6G), 10 μmol/L safranine T (ST), 0.1 mmol/L acridine red (AR), 0.1 mmol/L N,N′-dimethyl-3,4,9,10-perylenetetracarboxylic diimide (PTD), and 0.1 mmol/L tetra-(p-sulfonato phenyl) porphyrin (TPPS), 1 mmol/L vitamin C (Vc), 1 mmol/L hydrazine hydrate, 1.0 mmol/L hydroxylamine hydrochloride, 1 mmol/L H_2_O_2_, 1 mmol/L sodium sulfite, 1 mmol/L sodium borohydride and 0.1 mmol/L HAuCl_4_ were prepared. The silver nanorods (AgNR), silver nanochain (AgNC), blue silver nanotriangle (AgNT) and yellow silver nanotriangle (AgNP) were prepared by NaBH_4_, hydrate, H_2_O_2_-NaBH_4_ and NaBH_4_ reduction respectively (see SM). All reagents were of analytical grade and the water was doubly distilled.

### Pretreatment of samples

The vanadium pentoxide/nitric acid/sulfuric acid was selected to treat samples. In the digestion process, the organic mercury can be decomposed completely and inorganic mercury was not loss because the pentavalent vanadium fix effectively inorganic mercury under temperature 140 °C. However, when the digestion temperature was higher than 140 °C, pentavalent vanadium will turn to tetravalent vanadium, but the fixed ability of tetravalent vanadium are far below pentavalent vanadium. Therefore 100 °C was chosen as digestion temperature. Vanadium pentoxide was reduced to blue tetravalent vanadium end of the digestion process. The pretreatment of cosmetics sample was as follows^59^: a 0.5 g sample, 0.05 g vanadium pentoxide and 7 mL nitric acid were added into a 100 mL conical flask, the mixture was heated at 100 °C to boil with stirring by magnetic heated stirrer. A 8 mL sulfuric acid was added after cool down and stop the digestion until color turns to blue-green. A small amount of water was added after solution cooling, then continue heating to get rid of the nitrogen dioxide and neutralizing excess acid in solution. The sample solution was obtained after diluting to 100 ml. A 200 μL sample solution was used to analyze mercury content according to the procedure. Then, a known amount of HgCl_2_ was added into the sample solution to obtain the recovery.

## Additional Information

**How to cite this article**: Wang, Y. *et al.* Label-free SERS study of galvanic replacement reaction on silver nanorod surface and its application to detect trace mercury ion. *Sci. Rep.* **6**, 19650; doi: 10.1038/srep19650 (2016).

## Supplementary Material

Supplementary Information

## Figures and Tables

**Table 1:** Assignment of major SERS peaks of VBB in nanosilver sol substrate.

Peak of AgNR (cm^−1^)	Peak of AgNC (cm^−1^)	Peak of AgNT (cm^−1^)	Peak of AgNP (cm^−1^)	Vibration mode 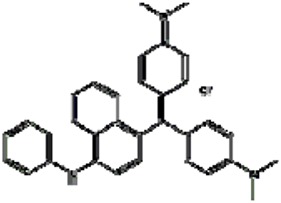
191 m	197 m	190 m	215 m	Skeletal bending
431 w	431 m	429 m	433 w	ρ(CH_2_)
677 w	677 w	676 w	675 w	γ(CH)
793 m	793 m	794 m	797 w	Ν(circle)
1164 s	1165 s	1172 s	1166 m	δ(CH_2_)
1199 s	1199 s	1199 s	1199 m	γ(NH_2_)
1360 s	1360 s	1362 s	1361 s	σ_as_(CH_2_)
1392 vs	1393 vs	1392 vs	1386 vs	δ(CH), (CH of C=C)
1445 m	1446 m	1452 m	1443 m	δ(CH_2_), δ_s_(CH_3_)
1478 m	1480 m	1481 m	–	δ(NH)
1562 s	1563 m	1563 m	1540 m 1563 m	σ(C-C) aromatic
1609 vs	1609 vs	1609 vs	1609 vs	σ(C=N) and σ(C=C)

^*^σ: stretching vibration; δ: bending vibration; δs: symmetric bending vibration; ν(circle): ring breathing; δ(circle): inner surface deformation of the ring; γ(CH): outside surface deformation of CH; γ(circle): outer surface deformation; ρ—rocking, in plane bending; γ—wagging.

Raman Intesity, vs: very strong; s: strong; m: medium; w- weak.

**Table 2:** Analysis features of the Hg(II)-nanosilver-VBB SERS systems.

System	Regression equation	Linear range (nmol/L Hg)	Correlation coefficient	Detection limit (nmol/L Hg)
Hg(II)-AgNR	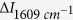 = 24.3*C* + 15.8	1.25–125	0.9955	0.2
Hg(II)-AgNC	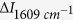 = 22.89*C* + 27	5–100	0.9920	3.8
Hg(II)-AgNT	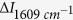 = 17.43C + 75	5–100	0.9926	3.6
Hg(II)-AgNP	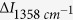 = 1.42*C* + 46	25–375	0.9942	21

**Table 3:** Analysis features of different molecular probe for detection Hg(II) in AgNR substrate.

Molecular probe	Regression equation	Linear range (nmol/L Hg)	Correlation coefficient	Detection limit (nmol/L Hg)
VBB	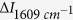 = 24.3*C* + 15.8	1.25–125	0.9955	0.2
ST	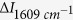 = 2.67*C* + 13	12.5–200	0.9958	11
Rh6G	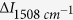 = 6.18*C* + 44	12.5–137.5	0.9942	4.5
RhS	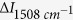 = 3.44*C* + 55	25–250	0.9925	11
RhB	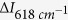 = 1.49*C* + 27	25–350	0.9904	17
AR	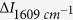 = 2.94*C* + 63	5–350	0.9940	8.4
PTD	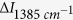 = 3.64 + 0.95	25–125	0.9908	5.5
TPPS	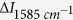 = 3.18 + 5.5	50–125	0.9989	15

## References

[b1] Syversen T. & Kaur P. The toxicology of mercury and its compounds. J. Trace Elem Med Bio. 26, 215–226 (2012). 22658719 10.1016/j.jtemb.2012.02.004

[b2] Zhang N., Li G., Cheng Z. & Zuo X. Rhodamine B immobilized on hollow Au–HMS material for naked-eye detection of Hg^2^^+^ in aqueous media. J. Hazardous Mater. 229, 404–410 (2012). 10.1016/j.jhazmat.2012.06.023 22771346

[b3] Hassan S. A., Moussa E. A. & Abbott L. C. The effect of methylmercury exposure on early central nervous system development in the zebrafish (Danio rerio) embryo. J. Appl. Toxicol. 32, 707–713 (2012). 21425300 10.1002/jat.1675

[b4] Erika E. *et al.* Molecular structure of mercury(II) thiocyanate complexes based on DFT calculations and experimental UV-electron spectroscopy and Raman studies. Spectrochim Acta A. 115, 574–582 (2013). 10.1016/j.saa.2013.06.072 23872016

[b5] Jiang J. *et al.* A sensitive colorimetric and ratiometric fluorescent probe for mercury species in aqueous solution and living cells. Chem. Commun. 48, 8371–8373 (2012). 10.1039/c2cc32867d 22798994

[b6] Yin C. H. *et al.* Sensitive determination of trace mercury by UV–visible diffuse reflectance spectroscopy after complexation and membrane filtration-enrichment. J. Hazardous Mat. 233/234, 207–212 (2012). 10.1016/j.jhazmat.2012.07.016 22831998

[b7] Campanella B., Onor M., Ulivo A. D., Giannarelli S. & Bramanti E. Impact of protein concentration on the determination of thiolic groups of ovalbumin: A size exclusion chromatography−chemical vapor generation-atomic fluorescence spectrometry study via mercury labeling. Anal. Chem. 86, 2251–2256 (2014). 24502672 10.1021/ac4041795

[b8] Quadros D. P. *et al.* Mercury speciation by high-performance liquid chromatography atomic fluorescence spectrometry using an integrated microwave/UV interface. Spectrochim Acta B. 101, 312–319 (2014).

[b9] Silva D. G., Portugal L. A., Serra A. M., Ferreira S. L. & Cerda V. Determination of mercury in rice by MSFIA and cold vapour atomic fluorescence spectrometry. Food Chem. 137, 159–163 (2013). 23200004 10.1016/j.foodchem.2012.10.019

[b10] Sommer Y. L. *et al.* Measurement of mercury species in human blood using triple spike isotope dilution with SPME-GC-ICP-DRC-MS. Anal. Bioanal. Chem. 406, 5039–5047 (2014). 24948088 10.1007/s00216-014-7907-4 PMC4685456

[b11] Pietila H. *et al.* Determination of methyl mercury in humic-rich natural water samples using N_2_-distillation with isotope dilution and on-line purge and trap GC-ICP-MS. Microchem. J. 112, 113–118 (2014).

[b12] Kim Y. H., Kim K. H., Yoon H. O. & Brown R. J. The application of gas chromatography-time-of-flight mass spectrometry to the analysis of monomethyl mercury at sub-picogram levels. Microchem. J. 110, 107–112 (2013).

[b13] Wang Z., Wu D., Wu G., Yang N. & Wu A. Modifying Fe_3_O_4_ microspheres with rhodamine hydrazide for selective detection and removal of Hg^2^^+^ ion in water. J. Hazardous Mater. 244–245, 621–627 (2013). 10.1016/j.jhazmat.2012.10.050 23177242

[b14] Lemos V. A. & Santos L. O. A new method for preconcentration and determination of mercury in fish, shellfish and saliva by cold vapour atomic absorption spectrometry. Food Chem. 149, 203–207 (2014). 24295696 10.1016/j.foodchem.2013.10.109

[b15] Wen G. Q., Liang A. H., Fan Y. Y., Jiang Z. L. & Jiang C. N. A Simple and rapid resonance scattering spectral method for detection of trace Hg^2^^+^ using aptamer-nanogold as probe. Plasmonics. 5, 1–6 (2010).

[b16] Liang A. H. *et al.* A highly sensitive resonance scattering spectral assay for Hg2^+^ based on the aptamer-modified AuRu nanoparticle-NaClO3- NaI-Cationic surfactant catalytic reaction. Anal. Lett. 44, 1442–1453 (2011).

[b17] Zhang Y., Liu Y., Zhen S. J. & Huang C. Z. Graphene oxide as an efficient signal-to-background enhancer for DNA detection with a long range resonance energy transfer strategy. Chem. Commun. 47, 11718–11720 (2011). 10.1039/c1cc14491j 21952343

[b18] Ding X. F. *et al.* Highly sensitive SERS detection of Hg^2^^+^ ions in aqueous media using gold nanoparticles/graphene heterojunctions. *ACS Appl*. Mat. Interfaces. 5, 7072–7078 (2013). 10.1021/am401373e 23855919

[b19] Muniz M. M., Pergolese B., Muniz M. F. & Caporali S. SERS effect from Pd surfaces coated with thin films of Ag colloidal nanoparticles. J. Alloys Compounds. 615, S357–S360 (2014).

[b20] Muller C. *et al.* Amnesic shellfish poisoning biotoxin detection in seawater using pure or amino-functionalized Ag nanoparticles and SERS. Talanta. 130, 108–115 (2014). 25159386 10.1016/j.talanta.2014.06.059

[b21] Shu L. *et al.* Highly sensitive immunoassay based on SERS using nano-Au immune probes and a nano-Ag immune substrate. Talanta. 123, 161–168 (2014). 24725879 10.1016/j.talanta.2014.02.015

[b22] Jiang X. H., Yang M., Meng Y. J., Jiang W. & Zhan H. J. Cysteamine-modified silver nanoparticle aggregates for quantitative SERS sensing of pentachlorophenol with a portable Raman spectrometer. ACS Appl. Mater. Interfaces. 5, 6902–6908 (2013). 23820578 10.1021/am401718p

[b23] Li P., Liu H. L., Yang L. B. & Liu J. H. Sensitive and selective SERS probe for Hg(II) detection using aminated ring-close structure of Rhodamine 6G. Talanta. 106, 381–387 (2013). 23598141 10.1016/j.talanta.2013.01.013

[b24] Kneipp K. *et al.* Single Molecule Detection Using Surface-Enhanced Raman Scattering (SERS). Phys. Rev. Lett. 78, 1667–1670 (1997).

[b25] Liu M. *et al.* SERS detection and removal of mercury(II)/silver(I) using oligonucleotide-functionalized Core/Shell magnetic silica sphere@Au nanoparticles. ACS Appl. Mater. Interfaces. 6, 7371–7379 (2014). 24738775 10.1021/am5006282

[b26] Senapati T. *et al.* Highly selective SERS probe for Hg(II) detection using tryptophan-protected popcorn shaped gold nanoparticles. Chem. Commun. 47, 10326–10328 (2011). 10.1039/c1cc13157e 21853207

[b27] Wang G. F. & Lipert R. J. Detection of the potential pancreatic cancer marker MUC4 in serum using surface-enhanced raman scattering. Anal. Chem. 83, 2554–2561 (2011). 21391573 10.1021/ac102829b PMC3315109

[b28] Chon H., Lee S., Son S. W., Oh C. H. & Choo J. Highly sensitive immunoassay of lung cancer marker carcinoembryonic antigen using surface-enhanced raman scattering of hollow gold nanospheres. Anal. Chem. 81, 813029–3034 (2009). 10.1021/ac802722c 19301845

[b29] Guo H. Y., Lu L. H., Wu C., Pan J. G. & Hu J. W. SERS tagged gold nanorod probes for immunoassay application. Acta Chim. Sin. 67, 1603–1608 (2009).

[b30] Ngo H. T., Wang H. N., Fales A. M. & Vo D. T. Label-free DNA biosensor based on SERS molecular sentinel on nanowave chip. Anal. Chem. 85, 6378–6383 (2013). 23718777 10.1021/ac400763c PMC4022286

[b31] Xu J. L. *et al.* Label-free detection of native proteins by surface-enhanced raman spectroscopy using iodide-modified nanoparticles. Anal. Chem. 86, 2238–2245 (2014). 24460183 10.1021/ac403974n

[b32] Barhoumi A., Zhang D., Tam F. & Halas N. J. Surface-enhanced Raman spectroscopy of DNA. J. Am. Chem. Soc. 130, 5523–5529 (2008). 18373341 10.1021/ja800023j

[b33] Barhoumi A. & Halas N. J. Label-Free Detection of DNA hybridization using surface enhanced raman spectroscopy. J. Am. Chem. Soc. 132, 12792–12793 (2010). 20738091 10.1021/ja105678z

[b34] Yang J. K. *et al.* Single-step and rapid growth of silver nanoshells as SERS-active nanostructures for label-free detection of pesticides. ACS Appl. Mater. Interfaces. 6, 12541–12549 (2014). 24988366 10.1021/am502435x

[b35] Zhang C. L., Luo L., Luo J., Evans D. G. & Sun X. A process-analysis microsystem based on density gradient centrifugation and its application in the study of the galvanic replacement mechanism of Ag nanoplates with HAuCl_4_. Chem. Commun. 48, 7241–7243 (2012). 10.1039/c2cc30457k 22421914

[b36] Skrabalak S. E. *et al.* Noble Metals on the nanoscale: optical and photothermal properties and some applications in imaging, Sensing, Biology, and Medicine. Acc. Chem. Res. 41, 1587–1595 (2008). 18447366 10.1021/ar7002804

[b37] Lu X. M., Chen J. Y., Skrabalak S. E. & Xia Y. M. Galvanic replacement reaction: a simple and powerful route to hollow and porous metal nanostructures. J. Nanoengineering and Nanosystems. 21, 1–17 (2008).

[b38] Sun Y. G., Mayers B. T. & Xia Y. M. Template-engaged replacement reaction: A one-step approach to the large-scale synthesis of metal nanostructures with hollow interiors. Nano Lett. 2, 481–485 (2002).

[b39] Liu G. L., Feng D. Q., Zheng W. J., Chen T. F. & Li D. An anti-galvanic replacement reaction of DNA templated silver nanoclusters monitored by the light-scattering technique. Chem. Commun. 49, 7941–7943 (2013). 10.1039/c3cc44126a 23900399

[b40] Bi Y. P. & Ye J. H. Heteroepitaxial growth of platinum nanocrystals on AgCl nanotubes via galvanic replacement reaction. Chem. Commun. 46, 1532–1534 (2010). 10.1039/b920497k 20162172

[b41] Netzer N. L., Tanaka Z., Chen B. & Jiang C. Y. Tailoring the SERS enhancement mechanisms of silver nanowire Langmuir-blodgett films via galvanic replacement reaction. J. Appl. Phy. 117, 16187–16194 (2013).

[b42] Ye W. C., Chen Y., Zhou F., Wang C. M. & Li Y. M. Fluoride-assisted galvanic replacement synthesis of Ag and Au dendrites on aluminum foil with enhanced SERS and catalytic activities. J. Mater. Chem. 22, 18327–18334 (2012).

[b43] Yang Y., Zhang Q., Fu Z. W. & Qin D. Transformation of Ag nanocubes into Ag-Au hollow nanostructures with enriched Ag contents to improve SERS activity and chemical stability. ACS Appl. Mater. Interfaces. 6, 3750–3757 (2014). 24476231 10.1021/am500506j

[b44] Pal A. *et al.* A. Galvanic replacement of As(0) nanoparticles by Au(III) for nanogold fabrication and SERS application. New J. Chem. 38, 1675–1683 (2014).

[b45] Fu J. J., Ye W. C. & Wang C. M. Facile synthesis of Ag dendrites on Al foil via galvanic replacement reaction with [Ag(NH_3_)_2_]Cl for ultrasensitive SERS detecting of biomolecules. Mater. Chem. Phy. 141, 107–113 (2013).

[b46] Yi Z. *et al.* Facile preparation of dendritic Ag–Pd bimetallic nanostructures on the surface of Cu foil for application as a SERS-substrate. Appl. Surface Sci. 258, 5429–5437 (2012).

[b47] Su L. *et al.* Highly sensitive surface-enhanced Raman scattering using vertically aligned silver nanopetals. RSC, Adv. 2, 1439–1443 (2012).

[b48] Jabeen S., Dines T. J., Withnall R., Leharne S. A. & Chowdhry B. Z. Surface-enhanced Raman scattering studies of rhodanines: evidence for substrate surface-induced dimerization. J. Phy. Chem. 11, 7476–7483 (2009). 10.1039/b905008f 19690722

[b49] Jiang Z. L. *et al.* A new silver nanorod SPR probe for detection of trace benzoyl peroxide. Sci. Rep. 4, 5327–5333 (2014). 24937042 10.1038/srep05323 PMC4060507

[b50] Yang Y., Liu J. Y., Fu Z. W. & Qin D. Galvanic replacement-free deposition of Au on Ag for core-shell nanocubes with enhanced chemical stability and SERS activity. J. Am. Chem. Soc. 136, 8153–8156 (2014). 24863686 10.1021/ja502472x

[b51] Wang G. Q. *et al.* Surface-enhanced raman scattering in nanoliter droplets: towards high-sensitivity detection of mercury (II) ions. Anal. Bioanal. Chem, 394, 1827–1832 (2009). 19444432 10.1007/s00216-009-2832-7

[b52] Martins R. *et al.* Identification of unamplified genomic DNA sequences using gold nanoparticle probes and a novel thin film photodetector. J. Non-crystalline Solids. 354, 2580–2584 (2008).

[b53] Ma P. Y. *et al.* Highly sensitive SERS probe for mercury(II) using cyclodextrin-protected silver nanoparticles functionalized with methimazole. Microchim. Acta. 181, 975–981 (2014).

[b54] Gao Y. *et al.* Direct determination of mercury in cosmetic samples by isotope dilution inductively coupled plasma mass spectrometry after dissolution with formic acid. Anal. Chim. Acta. 12, 6–11 (2014). 24491757 10.1016/j.aca.2014.01.002

[b55] Wang Y. Z. *et al.* Highly sensitive and specific determination of mercury(II) ion in water, food and cosmetic samples with an ELISA based on a novel monoclonal antibody. Anal. Bioanal. Chem. 403, 2519–2528 (2012). 22555680 10.1007/s00216-012-6052-1

[b56] Hamann C. R. *et al.* Spectrometric analysis of mercury content in 549 skin-lightening products: Is mercury toxicity a hidden global health hazard. J. Am. Acad. Dermatol. 70, 282–287 (2014). 10.1016/j.jaad.2013.09.050 24321702

[b57] Cobley C. M. & Xia Y. M. Engineering the properties of metal nanostructures via GRRs. Mater. Sci. Eng. R. 20, 44–62 (2010). 10.1016/j.mser.2010.06.002 PMC3003924 21180400

[b58] Gao S.Y., Jia X. X. & Chen Y. L. Old tree with new shoots: silver nanoparticles for label-free and colorimetric mercury ions detection. J. Nanopart. Res. 15, 1385 (2013).

[b59] GB/T 7917.1-1987. Health chemical standard of mercury in cosmetics. Beijing: Standards Press of China (1987).

