# Supplementary material for: Label-free SERS study of galvanic replacement reaction on silver nanorod surface and its application to detect trace mercury ion
Source: Sci Rep. 2016 Jan 21;6:19650. doi: 10.1038/srep19650 (PMC4726172; doi:10.1038/srep19650)
Supplement: Supplementary Information [file srep19650-s1.doc]

**Label-Free SERS Study of galvanic Replacement Reaction on Silver Nanorod Surface and Its Application to Detect Trace Mercury Ion**

Yaohui Wang^§^, Guiqing Wen^§^, Lingling Ye, Aihui Liang*, and Zhiliang Jiang*

Key Laboratory of Ecology of Rare and Endangered Species and Environmental Protection of Ministry Education, Guangxi Key Laboratory of Environmental Pollution Control Theory and Technology, Guangxi Normal University, Guilin 541004, China

**Keywords:** Hg(II); silver nanorod; galvanic replacement reaction; Victoria blue B; SERS quantitative analysis.

**Preparation of silver nanorods by NaBH_4_ reduction.** A 4.0×10^-4^ mol/L red silver nanorods (AgNR) was prepared as follows: a 46 mL water, 2ml 10mmol/L AgNO_3_, 3mL 60mmol/L trisodium citrate solution, 600µL 30%H_2_O_2_ and 600µL 0.1moL/L NaBH_4_ were added into a triangle flask in turn with stirring**,** when the color of the solution turn to deep red and put it into the microwave oven for 90 seconds with 2000 W power immediately. Then the AgNR with red color can be obtained after diluting to 50 mL.

**Preparation of silver nanochain by hydrazine hydrate reduction.** A 4.0×10^-4^ mol/L orange color silver nanochain (AgNC) was prepared as follows: a 45 mL water, 2ml 10mmol/L AgNO_3_, 3mL 60mmol/L trisodium citrate solution and 150µL 0.38mol/L hydrazine hydrate solution were added into a triangle flask in turn with stirring for 10 min**.** Then the AgNC with orange color can be obtained after diluting to 50 mL.

**Preparation of blue silver nanotriangle.** A 4.0×10^-4^ mol/L blue silver nanotriangle (AgNT) was prepared as follows: a 46 mL water, 2ml 10mmol/L AgNO_3_, 3mL 60mmol/L trisodium citrate solution, 600µL 30%H_2_O_2_ and 600µL 0.1moL/L NaBH_4_ were added into a triangle flask in turn with stirring**,** the AgNT solution was heated at 60 ℃ water bath for 15 min to remove excess H_2_O_2_. Then the AgNT with blue color can be obtained after diluting to 50mL.

**Preparation of silver nanoparticles*.*** A 1.8×10^-4^ moL/L yellow silver nanotriangle (AgNP) was prepared as follows: a 40 mL water, 3.5mL10g/L trisodium citrate and 385 μL 2.4×10^-2^ mol/L AgNO_3_ were added into a triangle flask in turn with stirring**.** Then a 4.0mL 0.5mg/mL NaBH_4_ was dripped slowly. After 20 min, the AgNP with yellow color can be obtained after diluting to 50ml.

Figure S1 SERS spectrum of the Hg(II)-AgNC-VBB system

1×10^-4^mol/L AgNC -75mmol/L HAc-20mmol/LNaCl -1.5×10^-7^mol/L VBB-Hg(II)

Figure S2 SERS spectrum of the Hg(II)-AgNT-VBB system

1×10^-4^mol/L AgNT -75mmol/L HAc-20mmol/LNaCl -1.5×10^-7^mol/L VBB-Hg(II).

Figure S3 SERS spectrum of the Hg(II)-AgNP-VBB system

4.5×10^-5^moL/L AgNP -75mmol/L HAc-20mmol/LNaCl -1.5×10^-7^mol/L VBB-Hg(II).

Figure S4 SERS spectrum of the Hg(II)-AgNR-ST system

1×10^-4^mol/L AgNR -75mmol/L HAc-20mmol/L NaCl -2.5×10^-6^mol/L ST- Hg(II).

Figure S5 SERS spectrum of the Hg(II)-AgNR-Rh6G system

1×10^-4^mol/L AgNR -75mmol/L HAc-6.5×10^-7^mol/L Rh6G- Hg(II).

Figure S6 SERS spectrum of the Hg(II)-AgNR-RhS system

1×10^-4^mol/L AgNR -75mmol/L HAc-1.3×10^-6^mol/L RhS-Hg(II).

Figure S7 SERS spectrum of the Hg(II)-AgNR-RhB system

1×10^-4^mol/L AgNR -75mmol/L HAc-1.25×10^-6^ mol/L RhB-Hg(II).

Figure S8 SERS spectrum of the Hg(II)-AgNR- AR system

1×10^-4^mol/L AgNR -75mmol/L HAc-2.5×10^-6^mol/L AR-Hg(II).

Figure S9 SERS spectrum of the Hg(II)-AgNR-PTD system

1×10^-4^mol/L AgNR -75mmol/L HAc-2.5×10^-6^mol/L PTD-Hg(II).

Figure S10 SERS spectrum of the Hg(II)-AgNR-TPPS system

1×10^-4^mol/L AgNR -75mmol/L HAc-2.5×10^-6^mol/L TPPS-Hg(II).

**Table S1 Assignment of SERS peaks of different molecular probes in AgNR sol**

| Molecular probes | Peak position/  peak strong | Vibration mode | Structure of molecular probe |
| --- | --- | --- | --- |
| ST | 273w | Skeletal bending | 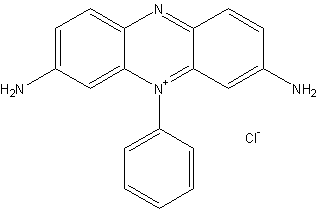 |
|  | 347s | ρ(CH) |  |
|  | 611vs | γ(CH) |  |
|  | 730w | ν( circle) |  |
|  | 750w | ν( circle) |  |
|  | 770w | ν( circle) |  |
|  | 828m | ρ(CH) |  |
|  | 1374s | σ_as_(CH_2_) aromatic |  |
|  | 1549s | σ(C-C) aromatic |  |
|  | 1641m | σ(C=N)and σ(C-C) aromatic |  |
| Rh6G | 226m | Ag-O | 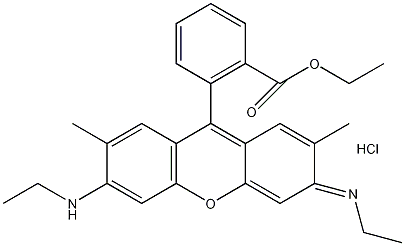 |
|  | 307m | ρ(CH) |  |
|  | 350m | ρ(CH) |  |
|  | 608s | δ(COO^-^) |  |
|  | 766m | ν( circle) |  |
|  | 1090w | σ_as_(C-O-C) |  |
|  | 1177 | γ(NH ) |  |
|  | 1308w | δ(C_6_H _5_─C) |  |
|  | 1359m | σ_as_(CH_2_) aromatic |  |
|  | 1508vs | δ(NH ) |  |
|  | 1570vs | σ(NH ) |  |
|  | 1647m | σ(C=N) and σ(C-C) aromatic |  |
| RhS | 255m | Ag-O | 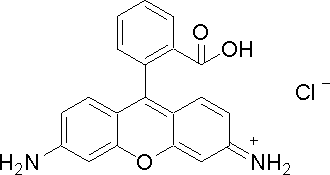 |
|  | 309m | ρ(CH) |  |
|  | 354m | ρ(CH) |  |
|  | 609s | δ(COO^-^) |  |
|  | 769m | ν( circle) |  |
|  | 1179m | γ(NH ) |  |
|  | 1309s | δ(C_6_H _5_-C) |  |
|  | 1360s | σ_as_(CH_2_) aromatic |  |
|  | 1508vs | δ(NH ) |  |
|  | 1571m | σ(NH ) |  |
|  | 1647s | σ(C=N) and σ(C-C) aromatic |  |
| RhB | 205m | Ag-O | 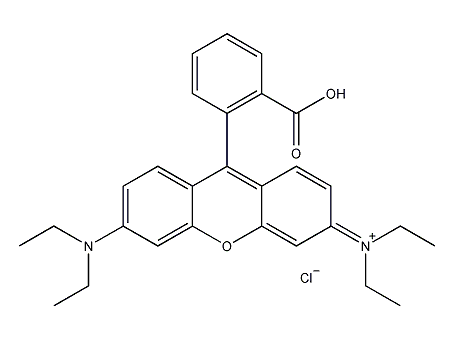 |
|  | 267m | ρ(CH) |  |
|  | 617vs | δ(COO^-^) |  |
|  | 931w | σ_s_(C-O-C) |  |
|  | 1079w | σ_as_(C-O-C) |  |
|  | 1195m | δ(CH_2_) |  |
|  | 1275s | δ(C_6_H _5_-C) |  |
|  | 1354s | σ_as_(CH_2_) aromatic |  |
|  | 1506s | δ(NH ) |  |
|  | 1524s | σ(NH ) |  |
|  | 1644s | Σ(C=N) and σ(C-C) aromatic |  |
| AR | 216m | Ag-O | 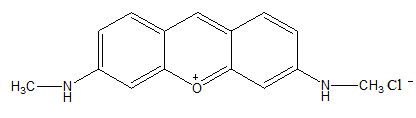 |
|  | 608s | γ(CH) σ(C-Cl) |  |
|  | 771m | ν( circle) |  |
|  | 1308m | δ(CH_2_) aromatic |  |
|  | 1358vs | σ_as_(CH_2_) aromatic |  |
|  | 1507vs | δ(NH ) |  |
|  | 1646s | σ(C-C) aromatic |  |
| PTD | 212vs | Ag-O | 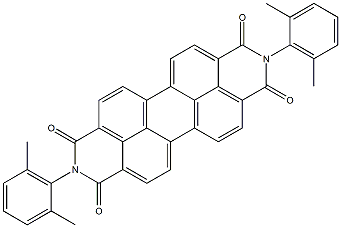 |
|  | 675s | γ( circle ) |  |
|  | 758m | ν( circle) |  |
|  | 795m | σ_as_(C-O-C) |  |
|  | 1172s | δ(CH_2_) |  |
|  | 1199m | δ(CH_2_) |  |
|  | 1361s | δ(CH_2_) |  |
|  | 1385vs | σ_as_(CH_2_) aromatic |  |
|  | 1587s | σ(NH ) |  |
|  | 1613s | σ(C-C) aromatic |  |
|  | 1986w | σ(C=O) |  |
| TPPS | 993s | SO^3-^ σ(CN) | 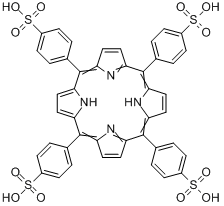 |
|  | 1173s | σ(C-N) |  |
|  | 1585vs | σ(NH ) |  |

**Table S2 Effect of foreign substances**

| Coexistent substance | Tolerance  Concentration  (µmol/L) | Relative error (%) | Coexistent substance | Tolerance  Concentration(µmol/L) | Relative error  (%) |
| --- | --- | --- | --- | --- | --- |
| Zn^2+^ | 10 | -1.2 | Mg^2+^ | 10 | 0.4 |
| Mn^2+^ | 10 | 7.1 | Pb^2+^ | 10 | -2.7 |
| Na^+^ | 10 | 1.2 | K^+^ | 10 | 2.1 |
| Ca^2+^ | 10 | 2.3 | Al^3+^ | 10 | 0.5 |
| Fe^3+^ | 10 | -1.1 | Ag^+^ | 10 | -8.8 |
| Cu^2+^ | 10 | 7.5 | Bi^3+^ | 5 | -3.6 |
| Br^-^ | 10 | -6.4 | I^-^ | 10 | 7.9 |
| TeO_4_^-^ | 10 | 3.3 | SeO_3_^2-^ | 8 | 0.5 |
| BSA | 4 | 4.7 | HSA | 1 | 7.8 |
| L-cystine | 4 | 3.6 | L-lysine | 1 | 8.2 |

**Table S3 Comparison of different analytical methods for detection mercury ion**

| Principle | LR(nmol/L) | DL(nmol/L) | Comments | Ref. |
| --- | --- | --- | --- | --- |
| AuNPs/rGO heterojunctions have been utilized for trace analysis of Hg^2+^ via T-Hg^2+^-T coordination. | 0.1-6000 | 0.1 | Highly sensitive but not accurate. | 18 |
| Hg^2+^ can combine with C=O and–NH_2_ of molecule probe, lead to the probe absorb on the surface of substrate decrease and SERS signals quenching | 1-10000 | 1 | Sensitive, rapid  but need two step | 23 |
| SERS-active platform by employing the oligonucleotide-functionalized magnetic silica sphere (MSS)@Au nanoparticles (NPs). This system exploits mismatched T-Hg-T bridges to capture Hg^2+^. | 0.1-1000 | 0.1 | Sensitive, but complicated. | 24 |
| Based on tryptophan protect flower gold nanoparticles can fast recognition Hg^2+^. | 25-2500 | 25 | Complicated but not Sensitive | 25 |
| Droplet-based microfluidics combined with surface-enhanced Raman scattering. | 0.5-2.5 | -- | Complicated. | 53 |
| Based on the binding of Hg^2+^ with T-T pairs results in AgNPs aggregation and the SERS signals increased. | 0-100 | 5 | Low sensitivity. | 54 |
| Using methimazole-functionalized and cyclodextrin-coated AgNPs as substrate, that exhibit strong SERS, and its intensity decreased with Hg(II). | 2.5-750 | 0. 5 | Rapid and sensitive | 55 |
| VBB exhibited strong SERS in AgNR sol, and GRR take places between AgNR with Hg (II) to form low SERS activity of Ag_core_-Hg_2_Cl_2shell_ that caused the SERS quenching. | 1.25-125 | 0.2 | Simple, rapid, accurate, sensitive. | This method |

**Table S4** Analysis results of Hg in cosmetic samples

| Sample | Single value (nmol/L) | Average (nmol/L) | Hg(II) content (*μ*g/g) | Added Hg(II)  (nmol/L) | Found Hg(II) (nmol/L) | Recovery (%) | RSD(%) |
| --- | --- | --- | --- | --- | --- | --- | --- |
| 1 | 45.01 49.81 50.39 48.87 50.14 | 48.84 | 196.41 | 50 | 94.87 | 96.55 | 3.65 |
| 2 | 50.59 48.58 50.06 45.95 43.20 | 47.68 | 191.65 | 50 | 95.38 | 95.40 | 3.72 |
| 3 | 35.89 35.76 33.71 34.86 32.81 | 34.61 | 139.13 | 50 | 84.82 | 100.41 | 1.39 |
| 4 | 7.91 8.74 9.84 9.19 8.69 | 8.88 | 35.72 | 50 | 62.66 | 107.56 | 0.58 |
| 5 | 13.46 14.94 12.76 13.83 13.62 | 13.72 | 55.25 | 50 | 64.5 | 101.56 | 0.67 |
| 6 | 19.87 22.66 22.99 20.57 18.51 | 20.92 | 84.10 | 50 | 71.66 | 101.48 | 1.73 |
